# Peer Review of “Determinants of Periodic Health Examination Uptake: Insights From a Jordanian Cross-Sectional Study”

**DOI:** 10.2196/71531

**Published:** 2025-02-05

**Authors:** Anonymous

**Keywords:** periodic health examination, PHE, preventive health services, routine health checkups, Jordan, cross-sectional study

*This is a peer-review report for “Determinants of Periodic Health Examination Uptake: Insights From a Jordanian Cross-Sectional Study.”*

## Round 1 Review

The following items were noted in this paper [1].

- Periodic health examination (PHE) uptake: Only 27.1% of participants underwent a PHE in the last 2 years.
- Predictors: Significant predictors include recent visits to a primary health care facility, monthly income, and knowledge about PHEs and preventive health measures.
- Nonsignificant factors: Gender, marital status, smoking status, and BMI did not show a significant association with PHE uptake.

### Strengths

1. Comprehensive analysis: The study employs a robust methodology, combining descriptive, inferential, and multivariate statistical techniques to provide a thorough understanding of PHE uptake.
2. Significant predictors identified: Key factors influencing PHE uptake were identified, offering valuable insights for health care providers and policy makers.
3. First of its kind in Jordan: This study fills a gap in existing knowledge by being the first to investigate PHE uptake in Jordan.

### Negative Points and Areas for Improvement

#### Cross-Sectional Design

- Limitation: The study’s design limits the ability to establish causality.
- Improvement: Future research could benefit from a longitudinal approach to better establish causal relationships between the identified predictors and PHE uptake.

#### Convenience Sampling

- Limitation: This method may introduce selection bias, and the online survey format may lead to measurement bias.
- Improvement: Employing a more randomized and stratified sampling method could enhance the representativeness and validity of the findings.

#### Limited Generalizability

- Limitation: Results may not be generalizable to populations outside of Jordan or those not included in the sample.
- Improvement: Expanding the study to include diverse populations and different geographic regions would provide a more comprehensive understanding of PHE uptake.

#### Survey Instrument

- Limitation: The questionnaire’s comprehensiveness and relevance to the Jordanian context might not have been fully ensured.
- Improvement: Pretesting the survey with a larger and more varied group, followed by adjustments based on feedback, could improve its applicability and accuracy.

#### Behavioral Factors

- Limitation: The study did not find a relationship between behavioral factors and PHE uptake, which contradicts findings in other contexts.
- Improvement: A more detailed investigation into cultural and societal influences on health behaviors in Jordan is needed to clarify these results.

#### English Language and Clarity

- Limitation: The manuscript contains some grammatical errors and awkward phrasings, which can detract from its readability.
- Improvement: A thorough review and editing for language and clarity by a native English speaker or professional editor would enhance the manuscript’s quality.

## Abbreviations

PHE: periodic health examination

## References

[1] Tayoun AA (2025). Determinants of periodic health examination uptake: insights from a Jordanian cross-sectional study. JMIRx Med.

